# A Parallel Computing Approach to Spatial Neighboring Analysis of Large Amounts of Terrain Data Using Spark

**DOI:** 10.3390/s21020365

**Published:** 2021-01-07

**Authors:** Jianbo Zhang, Zhuangzhuang Ye, Kai Zheng

**Affiliations:** School of Geography Information Engineering, China University of Geosciecnes, Wuhan 430074, China; Yezz@cug.edu.cn (Z.Y.); 20151002203@cug.edu.cn (K.Z.)

**Keywords:** spatial neighboring analysis, Spark, parallel computing, big data processing

## Abstract

Spatial neighboring analysis is an indispensable part of geo-raster spatial analysis. In the big data era, high-resolution raster data offer us abundant and valuable information, and also bring enormous computational challenges to the existing focal statistics algorithms. Simply employing the in-memory computing framework Spark to serve such applications might incur performance issues due to its lack of native support for spatial data. In this article, we present a Spark-based parallel computing approach for the focal algorithms of neighboring analysis. This approach implements efficient manipulation of large amounts of terrain data through three steps: (1) partitioning a raster digital elevation model (DEM) file into multiple square tile files by adopting a tile-based multifile storing strategy suitable for the Hadoop Distributed File System (HDFS), (2) performing the quintessential slope algorithm on these tile files using a dynamic calculation window (DCW) computing strategy, and (3) writing back and merging the calculation results into a whole raster file. Experiments with the digital elevation data of Australia show that the proposed computing approach can effectively improve the parallel performance of focal statistics algorithms. The results also show that the approach has almost the same calculation accuracy as that of ArcGIS. The proposed approach also exhibits good scalability when the number of Spark executors in clusters is increased.

## 1. Introduction

Spatial neighboring analysis, or focal statistics, is a raster-based spatial modeling method. It performs a neighborhood operation that computes an output raster, where the value for each output cell is a function of the values of all the input cells that are in a specified neighborhood around that location. The algorithms performed on the input raster include block statistics, filter, and terrain surface analysis (such as slope and aspect) and so on. They have been widely used in the estimation of surface water volume [1], prediction of tree growth [2], navigation reorientation [3], generation of digital terrain models [4], and some other fields.

Advances in data collection techniques (such as LiDAR and IfSAR) have brought us much more accessible three-dimensional mapping products in the form of digital elevation model (DEMs), and have also made the volume of digital terrain data reach an unprecedented scale. For instance, the global SRTM1 data with a resolution of one arc-second in latitude and longitude have $10^{11}$ points [5]. Since most commercial geographic information system software (such as ArcGIS) cannot handle the spatial neighboring analysis on this huge volume of data, an optimization approach for improving the computational efficiency of such focal algorithms is required. Generally, focal statistics employ overlapping neighborhoods to calculate a specified statistic for the cells within a moving window around each input cell. This procedure is iteratively executed and computationally intensive when massive terrain data are treated. In recent years, high-performance computing (HPC) has shown tremendous potential in parallelizing spatial data processing [6,7]. It is also capable of the challenge of accelerating the focal statistics algorithms.

Neighboring analysis applies to algorithms that are based on a fixed cell size and sliding analysis window, such as cellular automata [8], digital terrain analysis [9], and so on. Many research studies have been carried out on parallel implementation of raster-based spatial analysis via different parallel computing platforms. The Compute Unified Device Architecture (CUDA), OpenMP, MPI, and Intel’s Many Integrated Core (MIC) were developed to improve the processing speed of raster-based geocomputation [10,11,12]. To obtain suitable parallel performance, most research has focused on developing a parallel raster processing programming library, designing a set of parallel raster analytic operators, or achieving the best acceleration effect for a special algorithm over heterogeneous computing architectures [7,13,14].

In the last decade, distributed computing frameworks, like Hadoop MapReduce and Apache Spark, have been widely accepted for scalable processing of large spatial datasets. Existing solutions for big spatial data processing on Hadoop or Spark include Hadoop-GIS [15], Spatial Hadoop [16], Spatial Spark [17], and GeoSpark [18]. They contribute to the optimization of a spatial query (range or kNN) and join, spatial data indexing, and geometrical operations (e.g., overlap or intersect), which are often time-consuming and require a lot of preprocessing. In addition, the STARK framework [19] was presented especially for analyzing large spatio-temporal datasets. However, most of this research was focused on vector-based geocomputation over distributed computing architectures, such as Hadoop or Spark. Little work has been done to improve the execution performance of raster-based spatial analysis on Apache Spark with respect to design of data storage or implementation of the algorithm. Therefore, how one can make full use of the distributed computing environment to accelerate raster-based geospatial computation demands to be taken into consideration.

Spark has been widely accepted as the data processing platform for big datasets, and provides better performance than Hadoop MapReduce due to its large set of operators and support for cyclic data flow. However, Spark provides a general data model for processing any type of data, and does not provide native support for geo-raster spatial data. So, users have to implement special operators for raster-based data processing. Encouragingly, recent studies have shown that it is feasible to achieve desirable parallel acceleration for geo-raster spatial analysis using Spark. These research efforts to improve the computational efficiency of raster-based algorithms include the edge extraction from remote sensing images [20] and terrain viewshed analysis [21]. Nevertheless, few studies have been devoted to combining parallel approaches to spatial neighboring analysis with the Spark operation mechanism and principles.

In this article, a parallel computing approach implementing the slope algorithm (a quintessential example of focal statistics algorithms) using Spark is presented. First, we propose a tile-based data partitioning strategy to address the problem of utilizing the Hadoop Distributed File System (HDFS) to manage raster data. Then, we design a dynamic calculation window (DCW) computing strategy to optimize the data communication overhead brought by the halo phenomenon [22] of spatial neighboring analysis. Last, we introduce a data write-back and merging strategy to deal with persistent storage of massive tile statistics results. The implementation of spatial neighboring analysis includes the following three steps: (1) partitioning a raster file into multiple square tile files suitable for data organization of HDFS, (2) performing the slope algorithm on these tile data iteratively through integrated use of the parallel I/O and the communication optimization strategies, and (3) writing back the calculation results of all tiles and merging them into a whole slope result file.

The remainder of the article is organized as follows. Section 2 explains how focal statistics algorithms work, and the principles of the proposed strategies and the parallel implementation of the corresponding algorithms are described in detail. In Section 3, the computational experiments that were designed to evaluate the accuracy and performance of the Spark-based computing approach are presented. In Section 4, the results of the experiments are discussed. Section 5 contains some conclusions as well as some ideas for further work.

## 2. Methods

**Figure 1:**
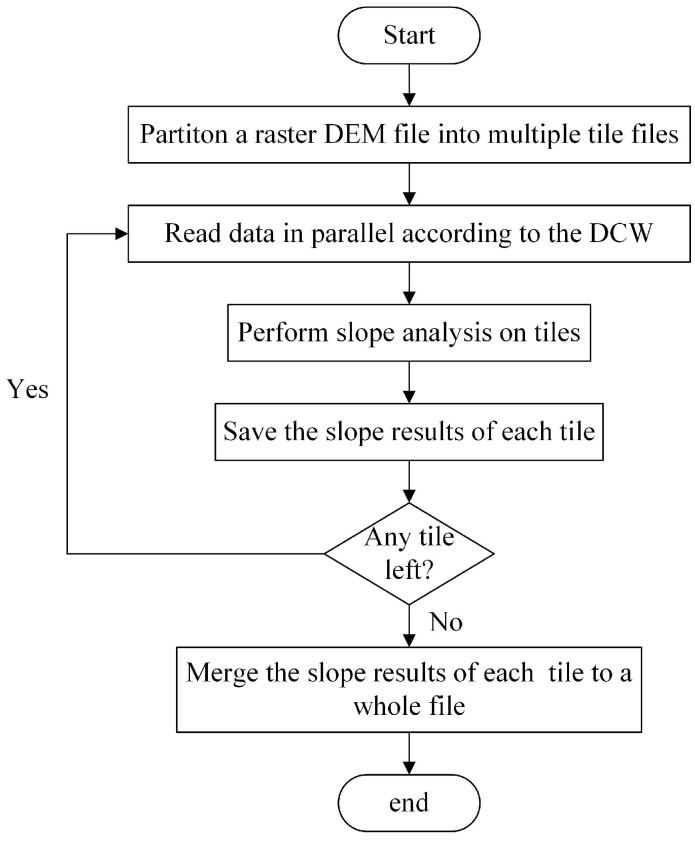
The flowchart of the neighboring analysis using Spark.

The distributed focal statistics algorithms synthesize a tile-based multifile storing strategy, a DCW computing strategy, and a write-back and merging strategy, which will be expatiated in the following. The flowchart of the proposed implementation of the neighboring analysis using Spark is illustrated in Figure 1.

### 2.1. Principles of Focal Statistics Algorithms

**Figure 2:**
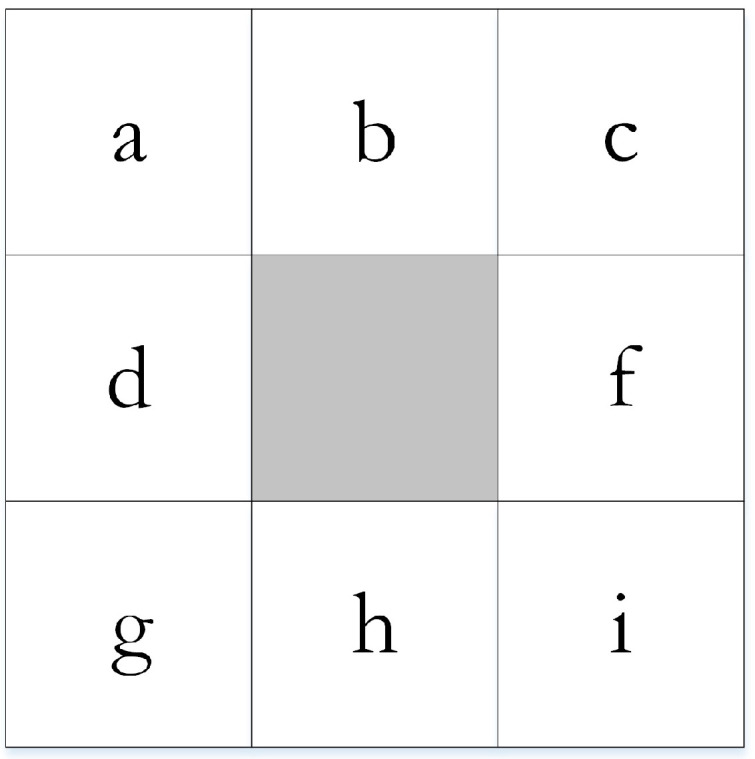
The square neighborhood of three by three cells.

Focal algorithms calculate a selected statistic for each input cell of the values within a specified neighborhood around it. For example, a square neighborhood is defined by eight points that are on a rectangle centered at a point for which a terrain topographic attribute (e.g., slope gradient) is to be computed from the elevation data. As each cell in the input is processed, the neighborhood is essentially a moving window that shifts along with it until an output raster dataset is produced. The size of the neighborhood specifically determines which cells surrounding the processing cell should be used in the calculation of each output value. The most typical neighborhood is a 3 × 3 cell window, as illustrated in Figure 2.

Slope is a commonly used topographic attribute for terrain analysis [23], which identifies the steepest downhill slope for a location on a surface. The slope algorithm takes an input surface raster and calculates an output raster containing the slope at each cell in a rectangular 3 × 3 cell analysis window. The Horn method [24] might be the most popular slope algorithm, as it has been adopted for the SLOPE function of ArcGIS. Thus, it was chosen as a representative of neighboring analysis in our parallel implementation. The operational principle of Horn’s slope algorithm is formulated as follows:

(1) $$slope_{degree}=\frac{180{}^{\circ}}{\pi }\times arctan\sqrt{\left(f_{x}\times f_{x}\right)+\left(f_{y}\times f_{y}\right)}$$

(2) $$f_{x}=\frac{\left(Z_{c}+2Z_{f}+Z_{i}\right)-\left(Z_{a}+2Z_{d}+Z_{g}\right)}{8\times xCellSize}$$

(3) $$f_{y}=\frac{\left(Z_{g}+2Z_{h}+Z_{i}\right)-\left(Z_{a}+2Z_{b}+Z_{c}\right)}{8\times yCellSize}$$

where $f_{x}$ and $f_{y}$ are the slopes in the *x* and *y* directions, respectively, $Z_{a}$, $Z_{b}$, $Z_{c}$, $Z_{g}$, $Z_{h}$, and $Z_{i}$ are the elevations of cells *a*, *b*, *c*, *g*, *h*, and *i*, respectively, and xCellSize and yCellSize are the cell sizes in the *x* and *y* directions, respectively.

**Figure 3:**
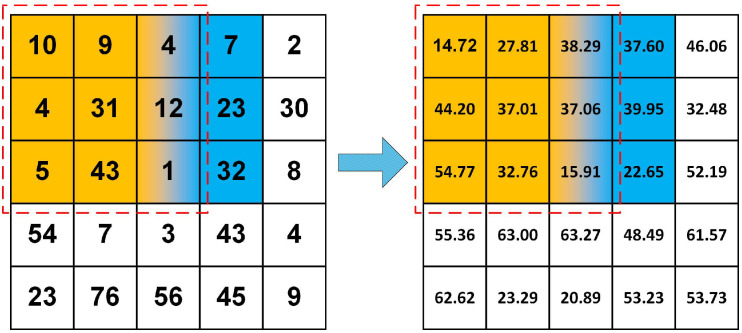
The calculation process of the slope algorithm.

After analyzing the implementation of the slope algorithm illustrated in Figure 3, we can summarize that: (1) The focal statistics algorithms could be accelerated by means of the power of parallel computing. They perform a given statistical calculation on each grid cell based on a sliding analysis window. The execution process is iterative, but the calculation results are independent; (2) there are a lot of overhead for raster data I/O operation when executing. For example, each grid cell must obtain the elevation values of its eight neighborhood points in a rectangular 3 × 3 cell analysis window in order to measure its slope value. The time complexity of this sequential algorithm is *O* (Row×Col), where Row and Col are the row and column numbers of the raster DEM. When massive amounts of high-resolution raster data are treated, overhead-intensive data access is inevitable.

In order to address these problems, three strategies were proposed to improve the parallel computing performance of the Spark-based focal statistics algorithms effectively.

### 2.2. Tile-Based Multifile Storing Strategy

**Figure 4:**
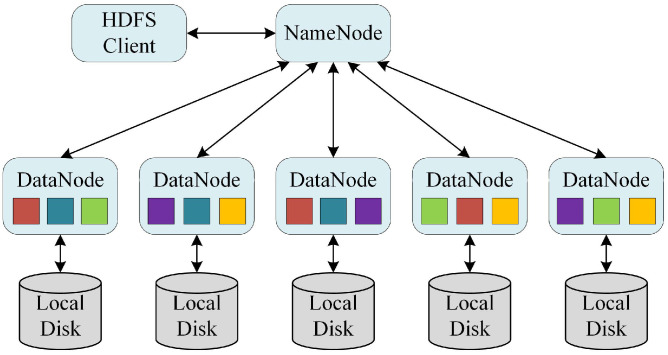
The overview of the Hadoop Distributed File System (HDFS).

The HDFS is an open-source distributed file system suitable for applications with high-throughput access requirements for large amounts of data. The HDFS has the concept of a block, but it is a much larger unit—128 MB by default. Like in a filesystem for a single disk, files in the HDFS are broken into block-sized chunks, which are stored as independent units [25]. Each HDFS block was replicated three times for fault tolerance, as illustrated in Figure 4. However, there is a problem with using the HDFS to store raster data directly. The calculation procedure of each cell requires its adjacent cells. If the raster data are submitted directly to the HDFS, the cell and its adjacent cells may be stored on different nodes, which could bring out additional communication overhead.

To address this problem, a storing strategy of HDFS divisions was proposed [26]. The tile-based partitioning method is commonly used for raster data partitioning, i.e., splitting large raster data into smaller grids with equal size. It is helpful to locate and access raster tiles during neighboring analysis. This partitioning method can cause a computation requested by an application to be executed as to near the data it operates on as possible, which helps minimize network congestion and increase overall system throughput [27]. Inspired by this method, this article proposed a tile-based multifile storing strategy. The storing strategy was designed to work in two steps: (1) partitioning the raster data into multiple tile files with equal size; and (2) submitting the tiles to the HDFS for persistent storage.

**Figure 5:**
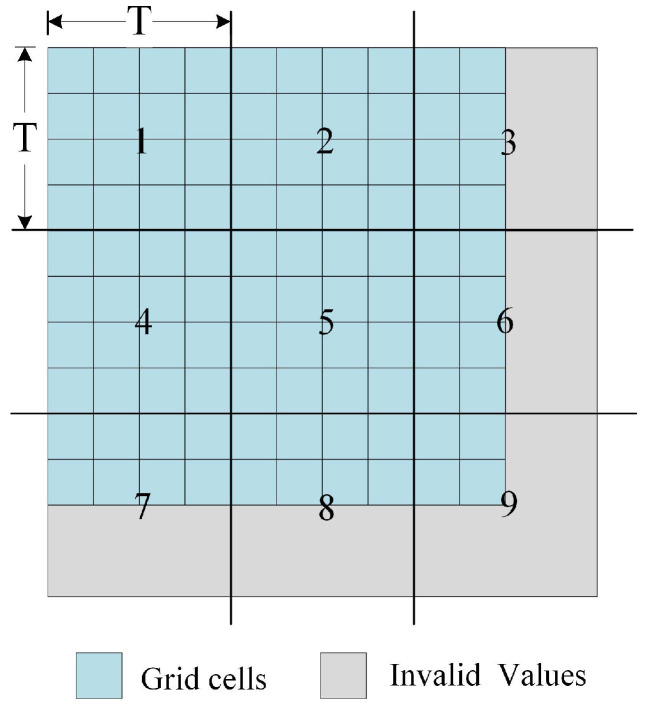
The tile partitioning strategy.

Determining the tile size of the proposed strategy is the key to solving the problem of across-HDFS division. The tile partitioning strategy is illustrated in Figure 5. The raster data will be divided according to tile size.

(4) $$TileSize=2^{n},1\leq n\leq log_{2}min\left(RowNum,ColNum\right)$$

where RowNum denotes the rows of the raster data and ColNum denotes the columns of the raster data. The raster data are divided by TileSize, and the part that is less than TileSize will be filled with an invalid value (such as −99,999) to ensure that each tile has the same size. Except for the volume of the raster data, the block size of the HDFS and the total memory size of the cluster should also be considered as affecting factors. In the following section, Section 4.2, 1024 × 1024 is proved to be the suitable size of a raster tile through experiments. This is because the byte length of a raster cell is often multiples of four bytes in most commercial geographic information system (GIS) software, such as four-byte floating-point- or integer-type data in ArcGIS. Therefore, the situation of one tile across two data blocks can be avoided.

The algorithm’s implementation of this storing strategy mainly includes the following steps:

(1) Get the metadata of the original raster file, and calculate the rows of tiles and columns of tiles using a predefined TileSize.

(5) $$TileRowNum=\left\{\begin{array}{cc} RowNum/TileSize+1 & \;if(RowNum\;mod\;TileSize\neq 0)\\ RowNum/TileSize & \;if(RowNum\;mod\;TileSize=0) \end{array}\right.$$

(6) $$TileColNum=\left\{\begin{array}{cc} ColNum/TileSize+1 & \;if(ColNum\;mod\;TileSize\neq 0)\\ ColNum/TileSize & \;if(ColNum\;mod\;TileSize=0) \end{array}\right.$$

**Figure 6:**
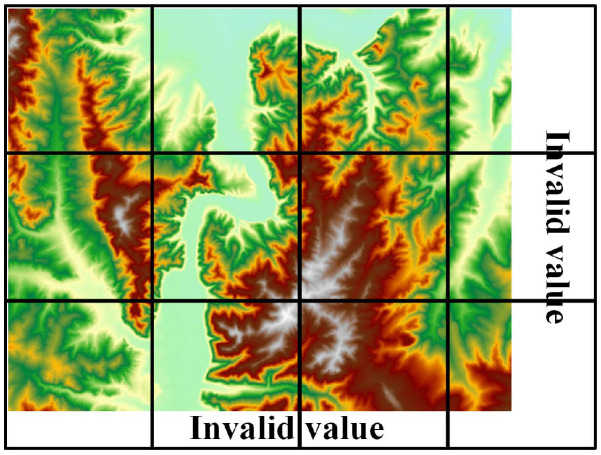
Method of partitioning raster data.

(2) Partition the raster file into multiple tile files according to the method shown in Figure 6. Note that when a tile lies in the last row or the last column, its blank data area needs to be filled with an invalid value.

(3) Write metadata information for subsequent calculations to a metadata file. Table 1 lists the included metadata.

(4) Upload the metadata file and multiple tile files to the HDFS.

### 2.3. Computing Strategy

**Figure 7:**
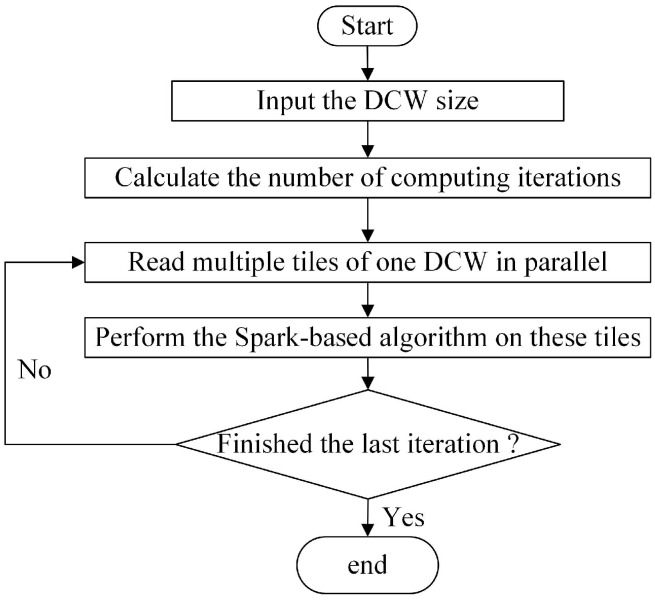
The flowchart of the computing strategy.

The computing strategy is comprised of the method for parallel reading of multiple tile files and the DCW computing strategy for the neighboring analysis. The flowchart of the proposed computing strategy is illustrated in Figure 7.

It firstly calculates the number of computing iterations according to the input DCW size. Then, it repeatedly reads the multiple tile files of each DCW window in parallel and performs the Spark-based slope calculation on them.

#### 2.3.1. The Method for Parallel Reading of Tile Files

The tile-based partitioning method could effectively solve the problem of utilizing the HDFS to store raster data, but also produces a great number of small tile files when processing large terrain data. Although having so many files introduces additional I/O reading costs, it provides the possibility to read data in parallel.

A resilient distributed dataset (RDD) is a fundamental data structure of Spark that expresses a directed acyclic graph (DAG) with RDD lineage. Spark provides transformations and actions to build the RDD lineage and explicitly expresses the algorithmic logic of applications [28]. In Spark, to initiate computation on worker machines, the driver process constructs a DAG that represents computation and dependency according to the requested RDD’s lineage. When a requested RDD has a long lineage, constructing computational dependencies can become a significant bottleneck. Such a problem can easily happen in streaming applications; for instance, when reading multiple tile files serially, a large number of transformations will be applied to generate the input RDD, which may run for a long period of time. In particular, when the RDD is lost, the re-computing of the long lineage will introduce considerable computational overhead [29]. Therefore, a proper reading method for multiple tile files through multiple tasks should be carefully considered to greatly reduce transformation operations and the risk of stack-overflow error.

**Figure 8:**
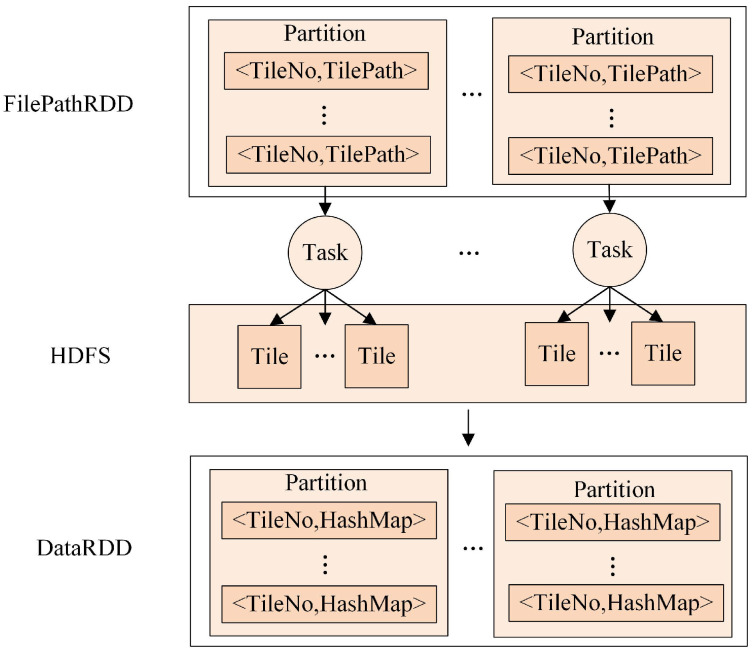
The parallel reading method for multiple tile files.

To address this problem, a parallel reading method for multiple tile files based on Spark was designed, as illustrated in Figure 8. The implementation of the method includes the following three steps: (1) getting the pathname set of multiple tile files generated by the above storing strategy; (2) producing a FilePathRDD of <TileNo,TilePath>, where TileNo denotes the serial number of a tile and TilePath denotes the tile path in HDFS; and (3) applying the map operation to transform the FilePathRDD into a new DataRDD of <TileNo,HashMap>, where HashMap is used to store all cell values of a tile. In step 3, the map operation is used to process each piece of data in a FilePathRDD in parallel, which makes it more efficient to read multiple tile files.

In Figure 8, when the map operation is applied to a FilePathRDD, each partition in the FilePathRDD will have a corresponding assigned task to complete the calculation. Then, these tasks will read multiple tile files in the HDFS in parallel. Eventually, a DataRDD will be formed to store the tile dataset as a basis for subsequent neighboring analysis.

#### 2.3.2. The DCW Computing Strategy

**(1)**  **The Definition and Implementation of the DCW**

Although the computing performance of raster-based analysis could be improved by using Spark, there are still some problems to be solved. For example, the traditional pixel-based calculating process brings frequent data I/O consumption, which also increases the scheduling time of the master node and the waiting time of the computing node. Therefore, the DCW computing strategy was proposed to accelerate the execution speed of the Spark-based slope algorithm. It adopts the DCW as a minimum computing unit and uses the raster tile as a minimum parallel task processing unit.

**Figure 9:**
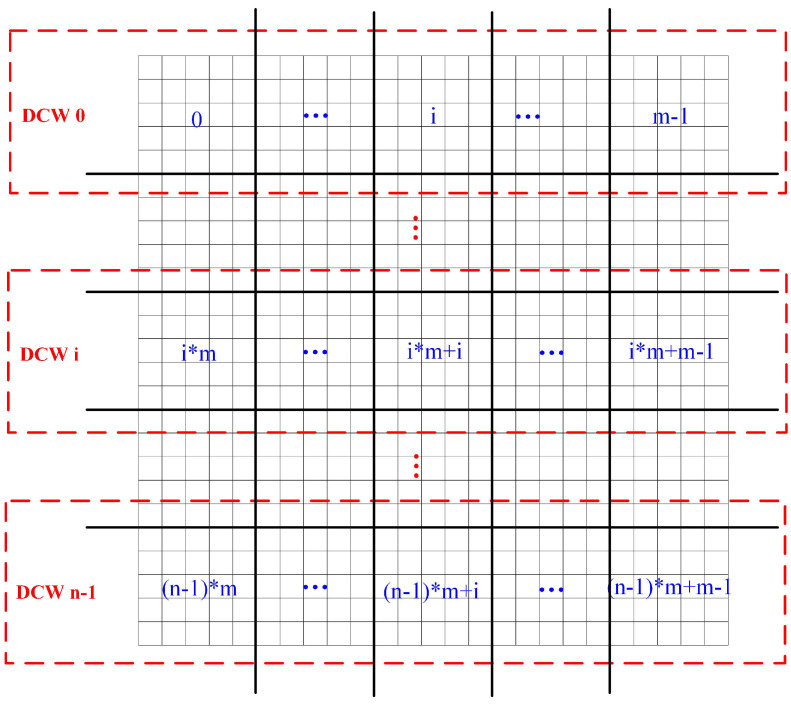
The forming principle of the DCW.

The DCW is defined as follows:

(a) If the upper-left corner of a tile is regarded as its location origin (latitude, longitude), a DCW refers to a tile dataset composed of one or more rows of tiles whose upper-left corners have the same latitude.

(b) The DCW size refers to the number of rows of tiles contained in the DCW. The forming principle of the DCW is illustrated in Figure 9.

**Figure 10:**
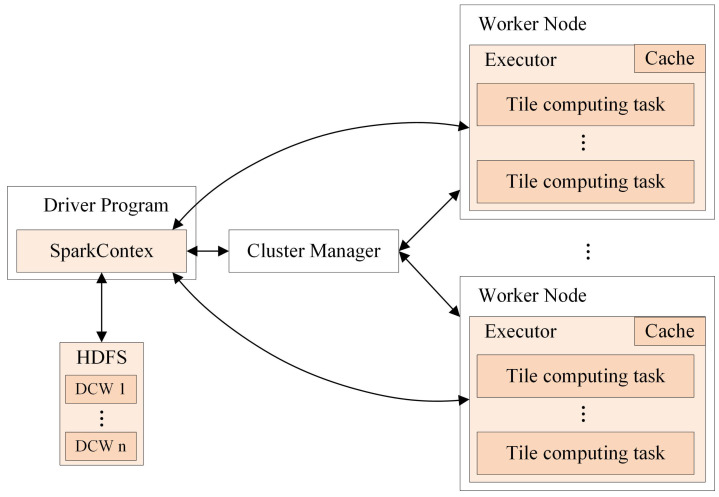
The dynamic calculation window (DCW) computing strategy in Spark.

Figure 10 illustrates how the DCW computing strategy works in Spark. Where *m* denotes the number of columns of tiles, *n* denotes the number of DCW. When a job is submitted, Spark will start a driver process and create SparkContext in the process. A SparkContext is the entry point for low-level API functionality. It takes on the task of creating RDDs, accumulators, and broadcast variables. In the proposed DCW computing strategy, SparkContext is in charge of reading multiple tile files to form a DataRDD. The DCW is regarded as the minimum computing unit of Spark job throughout the calculation process, and one DCW is used as input at a time. The calculation process based on the DCW is described as following. Firstly, SparkContext is utilized to construct a FilePathRDD that stores the pathname set of multiple tile files in one DCW. Then through reading these tile files in this FilePathRDD parallelly, SparkContext produces a DataRDD that stores all tiles of this DCW. Finally, SparkContext assigns computing tasks to each executor on distributed work nodes. These executors are responsible for performing the corresponding neighboring analysis based on the DataRDD.

More details of the Spark-based slope algorithm are described in Algorithm 1.

index denotes the index of each grid cell of a tile, value denotes the elevation of the cell, TileNo denotes the serial number of the tile, TilePath denotes the tile path in the HDFS, and TileColNum denotes the number of columns of tiles.

The Spark-based slope algorithm incorporates three main steps, as follows:

**Step 1: Read tile data in parallel**: In this step, the algorithm gets the pathname set of multiple tile files to construct the FilePathRDD, and applies the map operation named READDATA to transform the FilePathRDD into the DataRDD.

**Step 2: Calculate the data that need to be broadcast**: After reading the tile data, the algorithm selects a set of cells in each tile for other neighboring tiles to calculate, and broadcasts the cell set to each Spark worker node.

**Step 3: Filter the tiles to be calculated and calculate the slope value**: The algorithm enables each tile to acquire the cell set needed for neighboring analysis, and then the RDD of the slope analysis result is obtained by performing the map operation named DOSLOPE on the CalculateBlockRDD.
**Algorithm 1** Slope algorithm based on Spark**Input**: multiple tile files *T*, the size of DCW *S* **Output**: the slope analysis result *RDD* <*index*, *value*>  1: get the number of iterations based on *S*  2: get metadata information of DEM  3: N←numberofiterations  4: **function**
readData(TileNo,TilePath)  5:   Read the cell data of a tile  6: **end function**  7: **function**
select(TileNo,Hashmap<index,value>)  8:   Select the data to be broadcast  9: **end function** 10: **function**
doSlope(TileNo,Hashmap<index,value>) 11:   Perform neighboring analysis 12: **end function** 13: **for** each i∈N
**do** 14:   /* Step 1: Read tile data in parallel */ 15:   start←(i ∗ S−1) ∗ COLNUM 16:   DataRDD←DataRDD.filter(TileNo>=start) 17:   retrieve tiles by DCW in *T* 18:   get the pathname set of multiple tile files to construct FilePathSet<TileNo,TilePath> 19:   FilePathRDD<TileNo,TilePath>←parallelize(FilePathSet) 20:   DataRDD←DataRDD.union(FilePathRDD.map(readData)) 21:   /* Step 2: Calculate the data that need to be broadcast */ 22:   broadCastData= DataRDD.map( select).collectAsMap() 23:   criticalBroadCast=broadcast(broadCastData) 24:   /* Step 3: Filter the tiles to be calculated and calculate the slope value*/ 25:   low←i ∗ S ∗ COLNUM 26:   CalculateBlockRDD=DataRDD.filter(TileNo>=low) 27:   SlopeResultRDD = CalculateBlockRdd.map(doSlope) 28: **end for** 29: Output the SlopeResultRDD<TileNo,Hashmap<index,value>>

**(2)**  **The Solution to the Halo Phenomenon**

**Figure 11:**
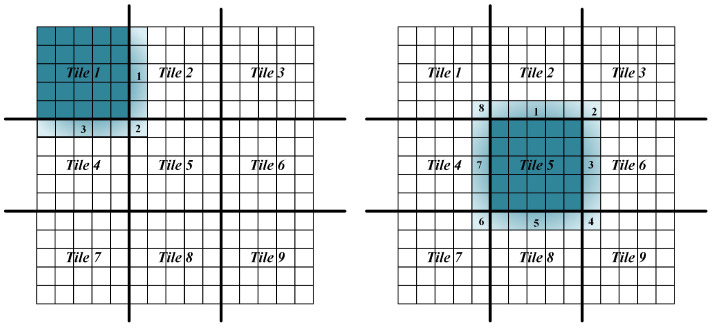
The halo phenomenon.

During the execution of the neighboring analysis, the computation of a tile requires not only its own cells, but also adjacent cells from other tiles. These extra cell data of other tiles form a halo around the original tile [14], which is called the halo phenomenon (illustrated as Figure 11). The halo phenomenon will bring inevitable communication overhead between computing tasks.

There are primarily two factors affecting the communication overhead in the DCW computing strategy. One is the tile size, and the other is the DCW size. The tile size affects the data size that needs to be broadcast. The smaller the tile size, the more data that need to be broadcast, and the time for network communication also increases. On the other hand, the DCW size affects the number of broadcasts of Spark broadcast variables. This number should be as small as possible to make the accumulated broadcast data as close to the number of cells that need to be broadcast as possible to reduce re-computing. Therefore, the DCW size should be as large as possible if cluster memory resources permit. These two factors will be further discussed in subsequent experiments.

In order to solve the halo problem in the DCW computing strategy, the total number of cells to be broadcast should be determined. Its calculation formula is defined as follows:

(7) $$TileRowNum=ROUNDUP\left(\frac{RowNum}{TileSize}\right)$$

(8) $$TileColNum=ROUNDUP\left(\frac{ColNum}{TileSize}\right)$$

(9) $$R=2\times TileSize\times \left(TileRowNum-1\right)\times TileColNum$$

(10) $$C=2\times TileSize\times \left(TileColNum-1\right)\times TileRowNum$$

(11) RepeatNum=4×TileRowNum×TileColNum

(12) N=R+C−RepeatNum

where RowNum and ColNum denote the rows and columns of the raster data, ROUNDUP denotes a function to round up, TileSize denotes the tile size, TileRowNum denotes the number of rows of tiles, TileColNum denotes the number of columns of tiles, *R* denotes the total number of cells to be broadcast in the row direction, *C* denotes the total number of cells to be broadcast in the column direction, RepeatNum denotes the number of cells to be counted repeatedly, and *N* denotes the number of cells that need to be broadcast.

### 2.4. Write-Back and Merging Strategy

In addition to data organization and computation, the persistent storage of the slope results is also important. After the calculation is completed, the results saved in memory in the form of tiles should be merged into a whole file on the HDFS. The problem of the sequence of data write-back and merging needs to be considered. This is because large data shuffles would be triggered if data merging operations are performed on Spark first, which may cause a lot of communication overhead and lead to memory overflow. Therefore, this article chose to firstly perform the data write-back strategy on Spark, and then to perform the data merging strategy on the HDFS.

**Figure 12:**
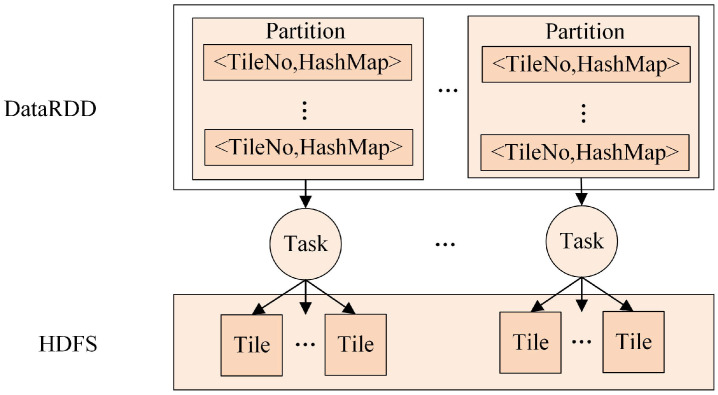
The data write-back strategy.

First of all, the goal of data write-back is to write the data back to the HDFS in parallel. The slope analysis results are stored in a local node after the calculation is completed. If the local node is a data node, the HDFS will use it to store the first copy. In this process, the tasks on different nodes do not affect each other. This is beneficial in improving the efficiency of data write-back operations. The implementation of the data write-back strategy is shown in Figure 12.

The main steps of the strategy include: (1) transforming the slope results from key-value RDDs into a byte array, (2) creating empty files named with TileNo, and (3) saving the byte array to its corresponding file.

In the second place, the goal of data merging is responsible for merging multiple tile files into a whole file. It can be regarded as the inverse process of data partitioning. In this process, the local position of each cell of a tile file is converted into the global position of the slope result file according to the following formula:

(13) $$Index=\left(\frac{TileNo}{TileColNum}\right)\;*\;TileSize\;*\;ColNum+i\;*\;ColNum+TileSize\;*\;\left(TileNo\%TileColNum\right)+j$$

where *i* and *j* denote the row and column numbers of a grid cell of a tile, TileNo denotes the serial number of a tile, TileColNum denotes the number of columns of tiles, TileSize denotes the size of a tile, and ColNum denotes the columns of all of the raster data.

**Figure 13:**
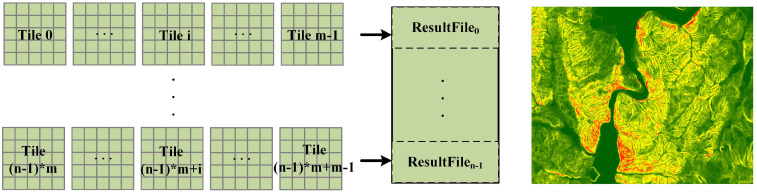
The data merging strategy.

The large number of I/O operations would directly affect the implementation performance during data merging. In order to improve the efficiency of data merging operations, a row/tile-based data merging strategy was designed, as shown in Figure 13.

More details of the implementation of the data merging algorithm are described in Algorithm 2.
**Algorithm 2** Data merging algorithm**Input**: multiple slope results’ tile files *T* **Output**: a whole slope result’s raster file *R*  1: create an empty file *R*  2: open *R* through output stream  3: **for** each i∈TileRowNum
**do**  4:   retrieve tiles in *T*  5:   open TileColNum file input streams in the i-th row of tiles  6:   **for** each j∈TileSize
**do**  7:    **for** each k∈TileColNum
**do**  8:     **if** i ×TileSize+j <= RowNum
**then**  9:      read TileSize cell data from the k-th input stream 10:      write the data to file **R** 11:     **end if** 12:    **end if** 13:   **end if** 14:   close the TileColNum file input streams. 15: **end if** 16: close the output stream of *R* 17: Output the file *R*


Here, TileRowNum denotes the number of rows of tiles, RowNum denotes the rows and columns of the raster data, TileColNum denotes the number of columns of tiles, and TileSize denotes the tile size.

## 3. Experiments and Results

### 3.1. Datasets

**Figure 14:**
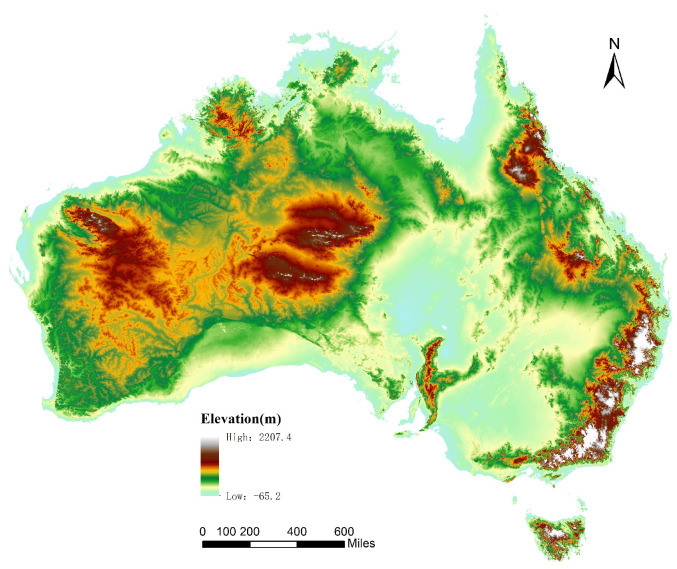
Elevation map of Australia.

The digital terrain data of Australia (https://data.gov.au/data/dataset/da926e47-1cd8-4dc9-b859-cbc18c29d858) were selected as the main data source. To evaluate the impact of the amount of data on the approach proposed in this article, four elevation datasets of different sizes were used to test the effectiveness of the proposed approach through data resampling. All dataset files listed in Table 2 were saved in extended GRD format (the Surfer grid file format of GoldenSoft). The elevation map is shown in Figure 14.

### 3.2. Execution Environment

A computer cluster with one name node and three data nodes was used as the hardware platform. These nodes were linked by 1 Gbps Gigabit Ethernet. There were, in all, 56 cores of CPU capacity, 200 GB of memory, and 22 TB of storage in the computing nodes of this cluster. The CentOS 6.5 operating system, Hadoop 2.7.1, and Spark 2.4.3 were used for each computer node. The corresponding program code was written in JDK-1.8 and Scala-2.10.4. Meanwhile, a workstation with ArcGIS 10.1 installed was utilized to execute the experimental algorithm to compare the computational accuracy with that of the cluster. It had eight cores of CPU capacity, 32 GB of memory, and 2 TB of storage.

### 3.3. Experimental Designs

In order to evaluate the method proposed in this article, four experiments were designed. Each experiment was repeated five times, and the results were averaged.

(1) To investigate the performance of the proposed calculation method, both the spatial analysis tool of ArcGIS on the workstation and the slope algorithm implemented on the cluster were executed using four datasets.

(2) To study the impact of the size of a raster tile and a DCW on the overall performance of the focal algorithms, the slope algorithm was also carried out on the four datasets with different tile sizes and DCW sizes.

(3) To verify the calculation accuracy of the Spark-based slope algorithm, the slope analysis tool of ArcGIS implemented on the workstation was chosen as a reference. They were both executed using four datasets to obtain the measurement results of accuracy.

(4) To test the scale-up performance of the proposed approach, the Spark-based slope algorithm was implemented with an incremental number of Spark executors.

### 3.4. Performance Evaluation

The accuracy metric was selected as an indicator to evaluate the calculation approach proposed in this article. It is defined as the mean and standard deviation [30] calculated from the statistical results of the subtraction of the two slope analysis raster files that resulted from the algorithm execution process performed on the cluster and the workstation. Its equations are as follows:

(14) $$\bar{X}=\frac{\sum_{i=1}^{n}X_{i}}{n}$$

(15) $$S=\sqrt{\frac{\sum_{i=1}^{n}{\left(X_{i}-\bar{X}\right)}^{2}}{n-1}}$$

where $\bar{X}$ is the mean, *S* is the standard deviation, and *n* is the number of raster grid cells.

### 3.5. Results

**Figure 15:**
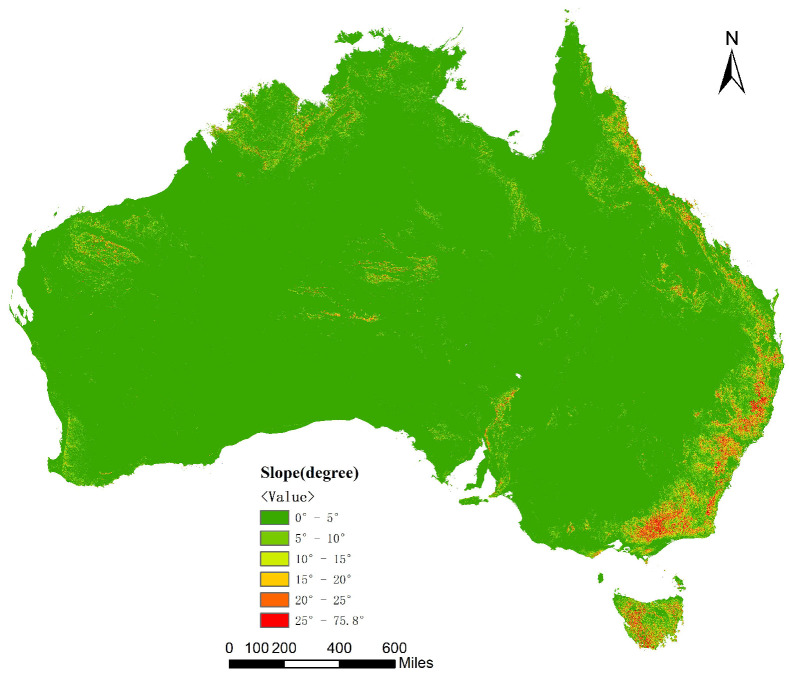
The slope map of the Spark-based algorithm.

The slope map of the experimental result is shown in Figure 15, which employed the Spark-based neighboring analysis.

## 4. Discussions

In Section 4.2, 1024 × 1024 was proved to be the optimal granularity for distributed neighboring computation with Spark. Therefore, in Section 4.1, Section 4.3, and Section 4.4, 1024 × 1024 was selected as the size of a raster tile. When computing such a large dataset as Grid4, the slope analysis tool of ArcGIS fails to obtain the slope results, as shown in following tables.

### 4.1. Effectiveness of the Approach on Parallel Performance

The computing performance results are shown in Table 3. Table 4 illustrates the execution time for each part of the proposed approach. The execution time of Spark here refers to the running time of Spark jobs. It mainly includes the communication overhead, the slope calculation time, and the result write-back time. The results show that as the amount of data grows, Spark-based slope algorithm performs better in computing efficiency. Firstly, the computing tasks of the Spark-based slope algorithm are based on the tile for parallel computing rather than a grid cell. This approach reduces the network communication between computing nodes and is more conducive to parallel computing. Secondly, as the amount of data increases, the computing performance of ArcGIS drops sharply, while the Spark-based slope algorithm performs well. This is due to the time cost in job initialization for the distributed framework occupying a high percentage of the overall computing time when the data size is relatively small. Finally, compared with the traditional GIS software, the proposed computing approach is capable of efficiently processing large amounts of data.

### 4.2. Factors Affecting the Computing Efficiency of the Approach

#### 4.2.1. The Tile Size

**Figure 16:**
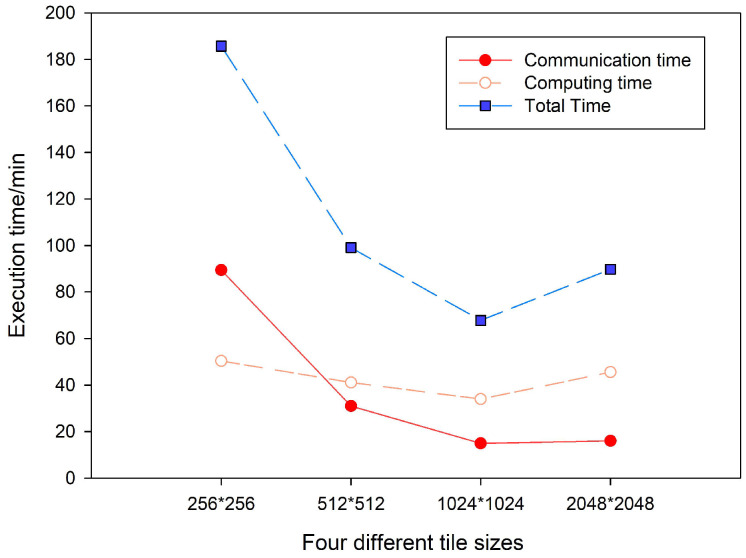
Comparison of the execution times using different tile sizes.

In this experiment, the slope algorithm was implemented using the Grid3 dataset. The time spent on communication and computing was recorded and compared using different tile sizes. The results for time consumption shown in Figure 16 indicate that the tile size has a direct influence on the parallelization performance. On the one hand, if the tile size is too small, more I/O operations and computing iterations will lead to a high communication overhead and scheduling time consumed by Spark. On the other hand, a large tile size requires more memory in the execution of computing tasks, which will increase the garbage collection (GC) time of JVM. A suitable tile size reduces the number of computing iterations and the GC time, thereby improving the total computing performance. From the point of view of the slope algorithm used in this experiment, a tile size of 1024 × 1024 offers optimal granularity for distributed computation under Spark.

#### 4.2.2. The DCW Size

**Figure 17:**
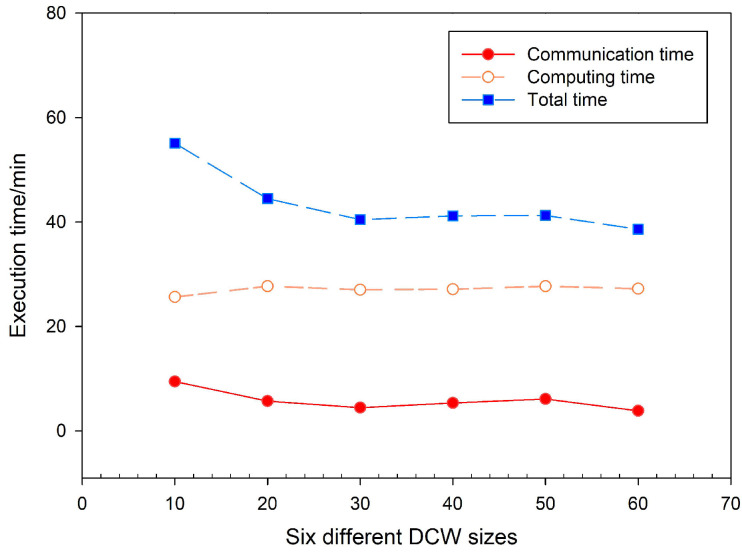
Comparisons of execution time using different DCW sizes.

In this experiment, the slope algorithm was again carried out using the Grid3 dataset. The time spent on communication and computing was recorded in six DCW sizes from 10 to 60. The results for time consumption in Figure 17 show that the DCW size also has an impact on the calculation performance. The DCW size determines the number of computational iterations, which may introduce scheduling time overhead for Spark. For example, the Grid3 dataset was partitioned into 60 rows of tiles and 73 columns of tiles with the size of 1024 × 1024. When the DCW size varies from 10 to 60, the number of computational iterations is, in turn, 6-3-2-2-2-1. Among them, the number of iterations for all the three DCW sizes (30, 40, and 50) is 2, and their total calculation time is roughly the same. The experimental results also demonstrate that the number of computational iterations is gradually decreased as the DCW size is increased. While limiting the number of iterations, the DCW computing strategy also reduces the waiting time of computing nodes for task assignment. It enables the Spark-based slope algorithm to achieve better computational performance.

### 4.3. Accuracy Measurement of the Approach

In this experiment, the slope analysis method of ArcGIS was selected as a reference because of its popular application in practice. The subtraction matrix of the calculation result of ArcGIS and that of the Spark-based algorithm were firstly obtained, and then the mean and the standard deviation were calculated according to the matrix. The experimental differences shown in Table 5 indicate that the accuracy of the two algorithms implemented on the first three datasets is almost the same. The results of the two implementations are slightly different due to data conversion. Four datasets used in all experiments were saved in the binary file format of the Surfer grid. They need to be converted into a text file in ArcInfo ASCII Grid format for ArcGIS rasters. There is an inevitable precision loss during this floating-point data conversion. This also directly leads to acceptable differences ($10^{-6}$) in the experimental results.

### 4.4. Scale-Up Performance of the Approach

**Figure 18:**
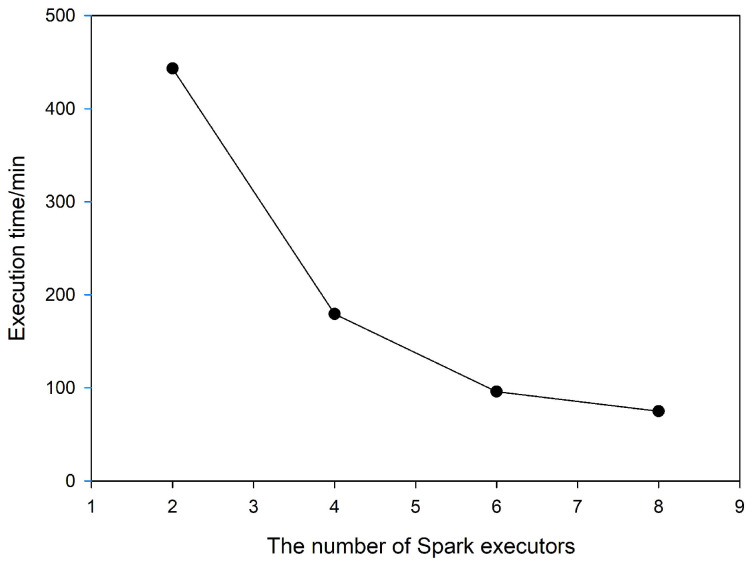
Comparison of the execution times of different numbers of executors.

In this experiment, the Spark-based slope algorithm was implemented using the Grid3 dataset with an incremental number of Spark executors. The execution time results shown in Figure 18 and Table 6 indicate that the proposed computing approach exhibits satisfactory scalability on the Spark clusters. The computing time decreases stably as the number of executors is increased. This is because more executors can better meet the computing requirement of data locality and improve parallelism of the calculations to achieve better performance.

## 5. Conclusions

Spatial neighboring analysis is an important component of raster-based geospatial analytics. Its execution efficiency is strongly influenced by the amount of raster data. Focal statistics algorithms can achieve desirable computational performance for processing large amounts of terrain data by exploiting the distributed computing capability of Apache Spark. In this article, we presented a Spark-based parallel computing approach for neighboring analysis algorithms, which integrates a tile-based multifile storing strategy, a dynamic calculation window (DCW) computing strategy, and a data write-back and merging strategy. First, the basic idea of the proposed tile-based multifile storing strategy is to take a raster tile as the minimum processing unit while performing the transformation and action functions of Spark, which is helpful in reducing the data transfer overhead of the distributed computation. Then, the DCW computing strategy is beneficial for optimizing communication overhead to improve the parallel performance of the neighboring analysis. Last, the proposed write-back and merging strategy contributes to the persistent storage of the massive analysis results. The experimental results showed that the Spark-based focal statistics algorithms dramatically reduced the computing time, achieved satisfactory performance and good accuracy, and scaled reasonably well as the data volume and the number of Spark executors of clusters increased.

In future work, we will first collect more high-resolution elevation data to further measure the parallel performance of the proposed computing approach. The experiment on the scale-up performance of the proposed approach was not sufficient due to the constraints of the cluster hardware environment. We will conduct the experiment in a cluster that is composed of better configured computational nodes. Thirdly, through the experiments, we found that the overhead of calculation was higher than that of the communication, and a proper DCW size should be determined based on the current cluster computing resources. We will also investigate how to further optimize our strategies to gain higher efficiency. Last but not least, existing geo-distributed computing frameworks presented in the literature (such as Hadoop-GIS, Spatial Hadoop, Spatial Spark, GeoSpark, and STARK) focus on big geo-vector data processing, and rarely involve geo-raster analysis. We will continue to search for some suitable geo-raster computational frameworks and conduct quantitative comparisons with them. 

## Figures and Tables

**Table 1:** Metadata Information.

Metadata Item	Numeric Type	Meaning	Bytes
TileSize	short	Byte size of a tile	2
ColNum	Integer	Columns of the raster	4
RowNum	Integer	Rows of the raster	4
xMin	double	Minimal x of the raster	8
xMax	double	Maximal x of the raster	8
yMin	double	Minimal y of the raster	8
yMax	double	Maximal y of the raster	8
zMin	double	Minimal z of the raster	8
zMax	double	Maximal z of the raster	8
TileColNum	Integer	Columns of tiles	4
TileRowNum	Integer	Rows of tiles	4
AddColNum	Integer	Columns of invalid values added	4
AddRowNum	Integer	Rows of invalid values added	4

**Table 2:** Australian elevation datasets.

Dataset	Size (GB)	Grid-Cell Size	Columns	Rows	Grid Cells
Grid1	2.69	5″ × 5″	29,520	24,481	722,679,120
Grid2	7.47	3″ × 3″	49,200	40,801	2,007,409,200
Grid3	16.8	2″ × 2″	73,800	61,201	4,516,633,800
Grid4	29.9	1.5″ × 1.5″	98,399	81,602	8,029,555,198

**Table 3:** Comparison of the execution times of ArcGIS and Spark (time unit: min).

Dataset	ArcGIS	Spark
Grid1	38.40	8.17
Grid2	116.89	17.85
Grid3	2444.05	37.33
Grid4	-	74.34

**Table 4:** The time spent on the three strategies (time unit: min).

Dataset	The Spark-Based Parallel Computing Approach			
	Strategy 1	Strategy 2	Strategy 3	
			Write-Back	Merging
Grid1	1.5872	7.21	0.96	2.13
Grid2	5.32	15.25	2.60	4.75
Grid3	10.78	31.73	5.60	8.77
Grid4	24.84	64.24	10.10	12.06

**Table 5:** The accuracy metrics of the Spark-based algorithm.

Dataset	Mean	Standard Deviation
Grid1	2.15 × 10^−6^	2.85 × 10^−6^
Grid2	3.57 × 10^−6^	4.74 × 10^−6^
Grid3	4.54 × 10^−6^	5.09 × 10^−6^
Grid4	-	-

**Table 6:** Time comparison of different numbers of executors (time unit: min).

Dataset	Number of Executors			
	2	4	6	8
Grid 3	443.2	179.31	95.85	74.91

## Data Availability

Data available in a publicly accessible repository.The data presented in this study are openly available in https://data.gov.au/data/dataset/da926e47-1cd8-4dc9-b859-cbc18c29d858.
